# A Bayesian quasi-likelihood design for identifying the minimum effective dose and maximum utility dose in dose-ranging studies

**DOI:** 10.1177/09622802241239268

**Published:** 2024-04-04

**Authors:** Feng Tian, Ruitao Lin, Li Wang, Ying Yuan

**Affiliations:** 1 Department of Biostatistics, 4002 The University of Texas MD Anderson Cancer Center, Houston, TX, USA; 2 Department of Statistics, AbbVie Inc., North Chicago, IL, USA

**Keywords:** Bayesian adaptive design, dose finding, risk–benefit tradeoff, phase II trials

## Abstract

Most existing dose-ranging study designs focus on assessing the dose–efficacy relationship and identifying the minimum effective dose. There is an increasing interest in optimizing the dose based on the benefit–risk tradeoff. We propose a Bayesian quasi-likelihood dose-ranging design that jointly considers safety and efficacy to simultaneously identify the minimum effective dose and the maximum utility dose to optimize the benefit–risk tradeoff. The binary toxicity endpoint is modeled using a beta-binomial model. The efficacy endpoint is modeled using the quasi-likelihood approach to accommodate various types of data (e.g. binary, ordinal or continuous) without imposing any parametric assumptions on the dose–response curve. Our design utilizes a utility function as a measure of benefit–risk tradeoff and adaptively assign patients to doses based on the doses’ likelihood of being the minimum effective dose and maximum utility dose. The design takes a group-sequential approach. At each interim, the doses that are deemed overly toxic or futile are dropped. At the end of the trial, we use posterior probability criteria to assess the strength of the dose–response relationship for establishing the proof-of-concept. If the proof-of-concept is established, we identify the minimum effective dose and maximum utility dose. Our simulation study shows that compared with some existing designs, the Bayesian quasi-likelihood dose-ranging design is robust and yields competitive performance in establishing proof-of-concept and selecting the minimum effective dose. Moreover, it includes an additional feature for further maximum utility dose selection.

## 1. Introduction

Phase II dose-ranging trials play a crucial role in drug development. They serve as the gateways to advancing promising doses of drugs to confirmatory phase III trials. Specifically, for identifying drug doses for treating non-life-threatening diseases, which is the focus of this article, healthy volunteers are enrolled in phase I trials to assess the drug’s safety and identify the maximum tolerated dose (MTD). Based on phase I data, a range of doses is selected for investigation in phase II dose-ranging trials to establish the proof-of-concept (PoC), estimate the therapeutic window, and select target doses for subsequent confirmative trials.^1^

Several adaptive designs have been developed for dose-ranging trials.^2,3^ Compared with conventional one-stage designs, adaptive dose-ranging trial designs are more flexible and efficient, as they allow for prospectively planned modifications of one or more aspects of the design based on interim data from the trial participants.^4^ Examples of interim modifications include adding or dropping doses, adaptively randomizing patients to beneficial doses, and selecting the dose–response model while accounting for model uncertainty. Berry et al.^5^ proposed an adaptive dose-ranging trial design based on a normal dynamic linear model to sequentially assign doses to new patients. Dragalin et al.^6^ proposed an adaptive dose-ranging trial design based on the D -optimality criteria to determine the number of doses and patients allocated to each dose. Bretz et al.^7^ proposed using the combined multiple comparison test and modeling process (MCP-Mod) to identify the minimum effective dose (MED) based on a continuous endpoint. Miller et al.^8^ introduced the idea of the “interesting part” of dose curves and applied the $I_{L}$ -criterion and c -optimality criterion under a Bayesian framework to detect the dose–response and find target doses, which is an approach that was later extended to dose-ranging trials.^3,9^ Ivanova et al.^10^ proposed a two-stage dose-ranging designs based on isotonic regression. Most existing dose-ranging trial designs focus on a continuous efficacy endpoint but do not consider toxicity for adaptive decisions and dose selection. Pinheiro et al.^3^ and Antonijevic et al.^11^ considered the use of safety data in simulation studies of dose-ranging trials, but in these designs, the safety data are mainly used to compute the probability of success in phase III trials rather than to determine dose assignment and selection.

There is increasing interest in incorporating benefit–risk consideration into the assessment and selection of doses in dose-ranging trials. In 2009, the U.S. Food and Drug Administration (FDA) released guidelines for performing benefit–risk assessments of new drug and biological products.^12^ Recently, the FDA launched Project Optimus to reform the dose optimization and dose selection paradigm in oncology drug development.^13^ Although Project Optimus focuses on oncology, its central message, that is, that dose selection should be based on a benefit–risk assessment, is also applicable to other therapeutic areas. Another important reason for considering toxicity in dose-ranging trials is that the doses selected for dose-ranging trials are often based on very limited phase I data (e.g. data from six patients treated at one dose). There is large uncertainty on the safety of the doses: a dose regarded as safe in a phase I trial may be actually toxic. For example, if one of six patients had toxicity at a certain dose, the 95% exact confidence interval for the underlying toxicity probability of that dose ranges from 0.004 to 0.641.

In addition, dose-ranging studies sometimes can have non-continuous efficacy endpoints, such as binary response. With the increasing availability and variety of biomarkers, there is a growing number of trials based on ordinal efficacy endpoints^14–19^ or semi-continuous bounded endpoints, such as a composite score.^20–23^ It’s desirable to devise a design that can accommodate diverse types of efficacy endpoints such as binary, ordinal, semi-continuous, and continuous endpoints. For example, Pinheiro et al.^24^ extended MCP-Mod to handle binary, count, or time-to-event endpoints.

In this article, we propose a Bayesian quasi-likelihood dose-ranging (BQD) design that can handle various types of efficacy endpoints and explicitly incorporates benefit–risk assessment into the decisions of dose assignment and selection. Our design employs a quasi-likelihood methodology as a unified framework to accommodate binary, ordinal, semi-continuous, and continuous endpoints. We utilize a utility function as a measure of benefit–risk tradeoff and to adaptively assign patients to doses based on the doses’ estimates of utility. The design takes a group-sequential approach. At each interim, the doses that are deemed overly toxic or futile are dropped. At the end of the trial, we use posterior probability criteria to assess the strength of the dose–response relationship (i.e. to establish the PoC). If the PoC is established, we identify both the MED and maximum utility dose (MUD) that maximizes the benefit–risk tradeoff.

Compared to existing methods, BQD has several appealing features. First, it does not make any parametric assumptions about the shapes of dose–toxicity and dose–efficacy curves and thus is robust. For comparison, MCP-Mod considers a set of prespecified candidate dose–efficacy models and uses model selection to improve the robustness of the design. However, as our simulation study showed, when the true dose–efficacy model is not in the candidate model set, the performance of MCP-Mod is subject to the influence of model misspecification. Second, BQD provides a uniformed framework to handle binary, ordinal, semi-continuous, and continuous endpoints. In contrast, most existing methods handle only one type of endpoint. Third, BQD explicitly accounts for benefit–risk assessment using utility and enables the selection of both the MED and MUD, whereas most existing methods focus on only efficacy and the MED. Last, owing to the use of a quasi-likelihood methodology with closed-form posteriors, the computation of BQD is easy and fast, which is important for application.

The remainder of this article is organized as follows. Section 2 introduces the probability model for safety and efficacy, the benefit–risk tradeoff, and the trial design. In Section 3, we use simulation to evaluate the operating characteristics of the BQD design. Section 4 gives a summary with concluding remarks.

## 2. Method

### 2.1. Probability model

Consider a dose-ranging trial with J doses, $d_{1}<\cdots <d_{J}$ , with $d_{1}$ denoting the placebo or control. For the i th patient, let $x_{i}$ denote a binary toxicity endpoint with $x_{i}=1$ indicating the occurrence of adverse events; $y_{i}$ denote the efficacy endpoint, which can be binary, ordinal, semi-continuous, or continuous; and $z_{i}$ denote the dose that the patient has received. Let $D_{\left(n\right)}=\{x_{i},y_{i},i=1,\ldots ,n\}$ represent the observed outcome data of the first n patients. We model the joint distribution of $\left(x_{i},y_{i}\right)$ by first specifying the marginal distribution of $x_{i}$ and then the conditional distribution of $y_{i}|x_{i}$ as follows.

We assume that $x_{i}$ , given a dose $d_{j}$ , follows a Bernoulli model:

$$\begin{array}{rl} x_{i}|\pi_{j} & ∼\mathrm{Bernoulli}(\pi_{j})\\ \pi_{j} & ∼\mathrm{Beta}(\alpha_{T_{j}},\beta_{T_{j}}) \end{array}$$

where $\pi_{j}$ is the toxicity probability of $d_{j}$ , and $\alpha_{T_{j}}$ and $\beta_{T_{j}}$ are hyperparameters that are often set as small values (e.g. 1) to obtain a vague prior. Let $n_{j}$ denote the number of patients treated at $d_{j}$ , and define $m_{j}=\sum_{i=1}^{n}x_{i}I(z_{i}=d_{j})$ as the number of patients who have experienced toxicity, where I(⋅) is the indicator function. Given $D_{\left(n\right)}$ , the posterior of $\pi_{j}$ arises as

(2.1) $$\pi_{j}|D_{\left(n\right)}∼\mathrm{Beta}(m_{j}+\alpha_{T_{j}},n_{j}-m_{j}+\beta_{T_{j}})$$

To impose the monotonicity of the dose–toxicity relationship, we adopt a modified isotonic regression method.^25^ Let $\left\{\pi_{j}^{\left(t\right)},t=1,\ldots ,T\right\}$ denote T (unconstrained) posterior draws from equation (2.1). This method applies the pool-adjacent-violators algorithm to $\left\{\pi_{j}^{\left(t\right)},t=1,\ldots ,T\right\}$ to obtain isotonically transformed posterior draws under the Bayesian framework that conforms to the monotonicity. When applying the pool-adjacent-violators algorithm, the weights for each dose are the reciprocal of the posterior variances of $\pi_{j}|D_{\left(n\right)},j=1,\ldots ,J$ . The estimation and inference of $\pi_{j}$ are based on the isotonically transformed posterior draws.

We next consider modeling the conditional distribution of $y_{i}|x_{i}$ . Let $\eta_{j,min}$ and $\eta_{j,max}$ respectively denote the minimum and maximum values of $y_{i}$ observed at $d_{j}$ . For bounded efficacy endpoints (e.g. $y_{i}$ is binary or ordinal), $\eta_{j,min}$ and $\eta_{j,max}$ are the lower and upper boundaries of $y_{i}$ , respectively. We first standardize $y_{i}$ to a value between [0,1] as follows:

$$\begin{array}{r} y_{i}^{*}=g(y_{i})=\frac{y_{i}-\eta_{j,min}}{\eta_{j,max}-\eta_{j,min}} \end{array}$$

We model $y_{i}|x_{i}$ using the quasi-Bernoulli likelihood method.^26^ According to this method, even though $y_{i}$ does not strictly adhere to a Bernoulli distribution (as it may take values other than 0 and 1), it can still be effectively modeled using a Bernoulli distribution, resulting in a quasi-likelihood, as follows:

$$\begin{array}{rl} y_{i}^{*}|x_{i}=k,z_{i}=d_{j} & ∼\mathrm{Quasi}\text{-}\mathrm{Bernoulli}(\theta_{jk})\\ \theta_{jk} & ∼\mathrm{Beta}(\alpha_{E_{jk}},\beta_{E_{jk}}) \end{array}$$

where $\theta_{jk}=E(y_{i}^{*}|x_{i}=k,z_{i}=d_{j})$ , and $\alpha_{E_{jk}}$ and $\beta_{E_{jk}}$ are hyperparameters that are often set as small values (e.g. 1) to obtain a vague prior. $y_{i}^{*}$ can be interpreted as the number of pseudo-events. McCullagh and Nelder^26^ demonstrated that under certain regulatory conditions, the quasi-Bernoulli likelihood produces consistent estimates while significantly simplifying computation due to its use of common distribution functions. This approach has been previously employed and has demonstrated effectiveness in phase I dose-finding designs.^28,27,29^

Let $s_{jk}=\sum_{i=1}^{n}y_{i}^{*}I(z_{i}=d_{j})I(x_{i}=k)$ denote the total number of pseudo-events at $d_{j}$ for total $n_{jk}$ patients with toxicity outcome $x_{i}=k$ , k=0,1 . Given $D_{\left(n\right)}$ , the posterior of $\theta_{jk}$ is given by

(2.2) $$\theta_{jk}|D_{\left(n\right)}∼\mathrm{Beta}(s_{jk}+\alpha_{E_{jk}},n_{jk}-s_{jk}+\beta_{E_{jk}})$$

Equation (2.2) provides the posterior distribution of $E(y^{*}|x_{i},d_{j}$ ). It is often useful to estimate $\mu_{j}=E(y_{i}|d_{j})$ , that is, the marginal efficacy probability of $d_{j}$ at the original scale of $y_{i}$ . This can be obtained as follows. Let $\left\{\theta_{j0}^{\left(t\right)},t=1,\ldots ,T\right\}$ and $\left\{\theta_{j1}^{\left(t\right)},t=1,\ldots ,T\right\}$ denote the posterior draws from the posterior distribution (2.2), and let $\left\{\pi_{j}^{\left(t\right)},t=1,\ldots ,T\right\}$ denote the posterior draws of $\pi_{j}$ from the posterior distribution (2.1). The posterior draws of $\mu_{j}$ can be obtained by marginalizing over $x_{i}$ as follows:

(2.3) $$\mu_{j}^{\left(t\right)}=\pi_{j}^{\left(t\right)}g^{-1}(\theta_{j1}^{\left(t\right)})+(1-\pi_{j}^{\left(t\right)})g^{-1}(\theta_{j0}^{\left(t\right)})$$

where $g^{-1}(\theta_{jk})=\theta_{jk}(\eta_{j,max}-\eta_{j,min})+\eta_{j,min}$ is the inverse of g(⋅) and transforms the conditional mean of $y^{*}$ back to its original scale. When appropriate, the isotonic regression transformation method described previously can be applied to $\left\{\mu_{j}^{\left(t\right)},t=1,\ldots ,T\right\}$ to impose the monotonicity on the dose–efficacy relationship. In our simulation, we focus on the case where the monotonicity assumption is applied, aligning with many dose-ranging designs (e.g. the Emax model) and the consideration that the dose–efficacy relationships are often monotonic in most disease areas.^30^ It is important to note that the proposed methodology does not require this assumption.

### 2.2. Benefit–risk tradeoff

Our design uses a utility function to measure the benefit–risk tradeoff between efficacy and toxicity. One simple and intuitive utility function is given by

(2.4) $$U_{j}=\mu_{j}-w\pi_{j}$$

where w is a pre-specified weight. According to this utility function, a one-unit increase in the toxicity rate leads to a w -unit penalty in the efficacy rate. For trials in which safety is a great concern, a larger value of w should be used. This utility function was considered previously by Ivanova et al.^31^ and Zhou et al.,^32^ and is a special case of the following more flexible benefit–risk tradeoff proposed by Liu and Johnson^33^:

$$\begin{array}{r} {\tilde{U}}_{j}=\mu_{j}-w_{1}\pi_{j}-w_{2}\pi_{j}I(\pi_{j}>\rho ) \end{array}$$

where ρ is a pre-specified threshold. Compared to utility (2.4), this utility function imposes an additional penalty of $w_{2}$ if the toxicity rate of $d_{j}$ is greater than that of the placebo by more than ρ . If $w_{2}=0$ , this utility function becomes (2.4); and if $w_{2}=\infty$ , we essentially require that the toxicity rate of $d_{j}$ cannot be ρ units higher than that of the placebo.

In addition to defining the benefit–risk tradeoff based on the marginal probability of toxicity and efficacy, such as (2.4), a more general approach involves assigning a utility score to each possible combination of patient-level toxicity and efficacy outcomes. The desirability of $d_{j}$ is then measured as the weighted average of these scores, weighted by their probability of occurrence. To illustrate, consider a binary toxicity and efficacy endpoints $x_{i}$ and $y_{j}$ , where there are four possible outcomes: ( $x_{i}=0,y_{i}=1$ ), ( $x_{i}=1,y_{i}=1$ ), ( $x_{i}=0,y_{i}=0$ ), and ( $x_{i}=1,y_{i}=0$ ). Let $a_{jk}$ denote the utility scores assigned to these outcomes, and define $q_{xy}(d_{j})=\mathrm{Pr}(x_{i}=x,y_{i}=y|d_{j})$ . The desirability of $d_{j}$ is calculated as $\sum_{x,y\in \{0,1\}}a_{xy}q_{xy}(d_{j})$ . For continuous $y_{j}$ , one can construct two utility functions $a_{0}(y)$ and $a_{1}(y)$ for patients with and without toxicity, respectively. Let $f(x,y|d_{j})=\mathrm{Pr}(x_{i}=x|d_{j})p(y_{j}=y|x_{i}=x,d_{j})$ be the joint probability function of $\left(x_{i},y_{i}\right)$ derived from our quasi-likelihood model, the desirability can be constructed similarly as $\int_{y}a_{0}(y)f(0,y|d_{j})\mathrm{dy}+\int_{y}a_{1}(y)f(1,y|d_{j})\mathrm{dy}$ . Lin et al.^29^ and Zhou et al.^32^ showed that this outcome-based utility approach encompasses the marginal-probability-based tradeoff (2.4) as a special case. Although our simulation focuses on the marginal-probability-based tradeoff, our methodology is directly applicable to this outcome-based tradeoff approach. To facilitate this flexibility, we opt to model the joint distribution of $\left(x_{i},y_{i}\right)$ , rather than their marginal distribution, in Section 2.1.

To safeguard patients from futility and/or a toxic dose, we define that a dose is admissible only if it satisfies the following safety and efficacy criteria:

(2.5) $$\begin{array}{rl} & \left(\mathrm{Safety}\right)\;\mathrm{Pr}(\pi_{j}>\pi_{1}+\bar{\pi }|D_{\left(n\right)})<C_{T} \end{array}$$

(2.6) $$\begin{array}{rl} & \left(\mathrm{Efficacy}\right)\;\mathrm{Pr}(\mu_{j}⩽\mu_{1}|D_{\left(n\right)})<C_{E} \end{array}$$

where $\bar{\pi }$ denotes the highest acceptable toxicity margin, and $C_{E}$ and $C_{T}$ are probability cutoffs that should be calibrated by simulation to obtain desirable operating characteristics. Depending on the tolerance level of safety and efficacy, $C_{E}$ and $C_{T}$ may range from 0.5 to 0.99. We use A to denote the set of admissible doses. Besides $d_{1}$ , only $d_{j}\in A$ can be used to treat patients during the trial.

### 2.3. Trial design

The BQD design takes a group sequential approach and consists of S stages. Let $c_{1},\ldots ,c_{S}$ denote the prespecified sample size of the S stages. The design is described as follows:

1. In the first stage, equally randomize $c_{1}$ patients into J arms.
2. For k=2,…,S stage, on the basis of the interim data, update the admissible dose set A and adaptively randomize $c_{k}$ patients to $d_{j}\in A$ . If A is empty, early stop the trial; no MED or MUD should be selected.
3. On the basis of the final data, assess whether a significant dose–response relationship exists to establish the PoC. If the PoC is established, select the MED and MUD; otherwise, claim that there is no significant dose–response relationship.

Below, we first describe the method for establishing the PoC and selecting the MED and MUD and then describe the procedure of adaptive randomization.

At the final analysis, we first assess the dose-response relationship as a PoC based on the following null and alternative hypotheses:

$$\begin{array}{r} H_{0}:\forall j\in \{1,\ldots ,J\},\;\mu_{j}⩽\mu_{1}\;\mathrm{versus}\;H_{a}:\exists j\in \{1,\ldots ,J\},\;\;\mathrm{such}\;\;\mathrm{that}\;\mu_{j}>\mu_{1} \end{array}$$

The PoC is established if at least one treatment arm meets the following criterion:

(2.7) $$\mathrm{Pr}(\mu_{j}>\mu_{1}|D_{\left(n\right)})>C_{\mathrm{PoC}}$$

where $C_{\mathrm{PoC}}$ is the probability cutoff. The value of $C_{\mathrm{PoC}}$ should be calibrated and determined by simulation under the null hypothesis to control the familywise type I error rate at a pre-specified level. If no dose arm meets the criterion (2.7) for establishing the PoC, we claim that there are no significant dose–response relationship; otherwise, we proceed to select the MED and MUD.

Specifically, we select the MED as the dose whose posterior mean of $\mu_{j}$ (denoted as $\hat{\mu_{j}}$ ) is the closest to $\hat{\mu_{1}}+\Delta$ among admissible doses, where Δ is the pre-specified clinically relevant difference for efficacy, that is,

(2.8) $$MED=argmin_{j\in A}|\hat{\mu_{j}}-(\hat{\mu_{1}}+\Delta )|$$

The selection of the MUD is based on the benefit–risk tradeoff quantified by utility. Let $\hat{U_{j}}$ denote the estimate of $U_{j}$ based on the final trial data. The MUD is selected as

(2.9) $$MUD=argmax_{j\in A}(\hat{U_{j}})$$

The proposed framework is flexible and can accommodate various definitions of the target dose based on toxicity and efficacy. For some applications, when desirable, we could impose an additional condition of MUD≥MED .

We now discuss the adaptive randomization rule in step 2 of the BQD design. Given the interim data $D_{\left(n\right)}$ , let $p_{1j}=\mathrm{Pr}(d_{MED}=d_{j}|D_{\left(n\right)})$ and $p_{2j}=\mathrm{Pr}(d_{MUD}=d_{j}|D_{\left(n\right)})$ denote the posterior probabilities that dose j is the MED and MUD, respectively. We assign patients based on the probabilities of a dose being the MED and MUD as follows:

(2.10) $$p_{j}\propto \tau p_{1j}^{\nu }+(1-\tau )p_{2j}^{\nu },\;j\in A$$

where τ is the prespecified weight that allows the researchers to prioritize the MED or MUD based on the trial objectives, and ν≥0 controls the adaptiveness of the subsequent randomization with ν=0 indicating equal randomization. For example, if the primary objective of the trial is to identify the MED (or the MUD), we can set τ=1 (or 0). If the primary objective is to identify both the MED and MUD, which is the focus of this article, we can set τ=1/2 . For dose $d_{j}\notin A$ , $p_{j}=0$ . In (2.10), $p_{1j}$ and $p_{2j}$ are calculated as the proportions of posterior draws of toxicity and efficacy model parameters (i.e. $\pi_{j}$ ’s and $\theta_{jk}$ ’s) that result in $d_{j}$ as the MED and MUD, respectively. The details are provided in the Supplemental Material (Table S6). We set ν=1 throughout the article.

To ensure reasonable power for establishing the PoC, it is important to assign a sufficient but not excessive number of patients to the control. Let $p^{\max}=\max\{\{p_{1j}\},\{p_{2j}\}\}$ . We assign patients to the control based on the following probability:

(2.11) $$p_{1}\propto \min(p^{\max},1/(J-1))$$

That is, the probability of assigning patients to the control equals the maximum randomization probability of the treatment arms or, if that maximum randomization probability is too high, 1/(J−1) . The latter rule is used to avoid allocating too many patients to the control. Accordingly, in (2.10), we adjust the randomization probability to the dose with the maximum $p_{1j}$ and the dose with the maximum value of $p_{2j}$ to $p^{\max}$ so that the expected MED and MUD can both have relatively high randomization probabilities. If identifying the MED (or the MUD) is the primary objective, we set τ=1 (or 0) and adjust $p^{\max}=\max\{p_{1j}\}$ (or $\max\{p_{2j}\}$ ).

When implementing the BQD design, a practical consideration is the determination of the total sample size and interim times. The total sample size (i.e. $c_{1}+\cdots +c_{S}$ ) can be selected through simulation to ensure reasonable power (e.g. 80%–90%) for PoC and accuracy in selecting the MED and MUD. An effective approach is to use a simple method, such as the ANOVA method, to establish an initial sample size and subsequently calibrate it through simulation. To ensure reliable interim decisions, it is advisable to choose a reasonably large $c_{1}$ (i.e. the sample size of the first interim) to ensure a reasonably accurate estimate of toxicity and efficacy. The remaining interims can be equally split for the remaining sample size. The choice of interims should balance logistical complexity and statistical performance and often vary across trials.

## 3. Simulation

### 3.1. Setup

**Figure 1.**
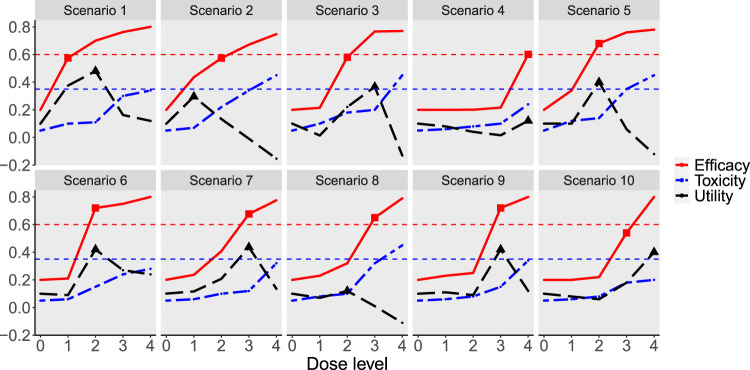
Simulation scenarios for the continuous endpoint. The solid red line is the dose–efficacy curve. The dash-dotted blue line is the dose–toxicity curve. The dashed black line is the dose–utility curve. The horizontal dashed red line corresponds to the target efficacy, and the horizontal dashed blue line is the maximum acceptable toxicity rate. The minimum effective dose is marked by a square ( ◼ ), and the maximum utility dose is marked by a triangle ( ▴ ).

We used simulation to evaluate the operating characteristics of the BQD design and compared them with those of a conventional analysis of variance (ANOVA) approach^2^ and MCP-Mod.^7^ MCP-Mod is based on five dose-efficacy models, including $E_{\max}$ , linear, linear in log-dose, logistic, and exponential models. We considered J=5 arms with $\left\{d_{1},\ldots ,d_{5}\}=\{0,1,2,3,4\right\}$ , where $d_{1}$ is a placebo. We assumed binary toxicity endpoint $x_{i}$ and considered continuous, ordinal, and binary efficacy endpoint $y_{i}$ . For the continuous endpoint $y_{i}$ specifically, the maximum sample size was N=200 enrolled in S=5 stages with cohort size $c_{1}=100$ , $c_{2}=\cdots =c_{5}=25$ . We used a latent-variable approach to generate the joint distribution of $\left(x_{i},y_{i}\right)$ . Let ${\tilde{x}}_{i}$ denote a latent continuous variable that is related to $x_{i}$ as $x_{i}=1$ if $\Phi^{-1}({\tilde{x}}_{i})>1-\pi_{j}$ ; otherwise, let $x_{i}=0$ , where $\Phi^{-1}(\cdot )$ is the inverse cumulative distribution function for the standard normal distribution. We generated $\left({\tilde{x}}_{i},y_{i}\right)$ based on the following bivariate normal model:

(3.1) $$\left(\begin{array}{c} {\tilde{x}}_{i}\\ y_{i} \end{array}\right)|\;z_{i}=d_{j}\overset{i.i.d}{∼}N\left(\left(\begin{array}{c} 0\\ \mu_{j} \end{array}\right),\left(\begin{array}{cc} 1 & \rho \sigma \\ \rho \sigma & \sigma^{2} \end{array}\right)\right)$$

where σ is the standard deviation of $y_{i}$ , and the correlation coefficient ρ induces correlation between ${\tilde{x}}_{i}$ (and thus $x_{i}$ ) and $y_{i}$ . We set ρ=0.3 and σ=1 such that the ANOVA approach has around 70% – 80% power for establishing the PoC in most scenarios considered. The mean efficacy of the placebo arm was $\mu_{1}=0.2$ , the clinically relevant effect was Δ=0.4 , and the maximum acceptable toxicity rate change was $\bar{\pi }=0.3$ . We varied the values of $\mu_{j}$ and $\pi_{j}$ to construct 10 representative dose–efficacy and dose–toxicity curves with different locations of the MED and MUD (Figure 1 and Table 1). To evaluate the robustness of the designs, we generated scenarios 1–4 based on parametric models, where MCP-Mod is correctly specified, and arbitrarily generated scenarios 5–10, for which the parametric models assumed that MCP-Mod may not hold. The models used to generate scenarios 1–4 which are motivated by Bretz et al.^7^ are provided in the Supplemental Material (Table S1).

**Table 1.** Simulation scenarios for the continuous efficacy endpoint.

	Dose levels					Dose levels				
	0	1	2	3	4	0	1	2	3	4
	Scenario 1					Scenario 2				
Mean eff.	0.20	**0.57**	0.70	0.76	0.80	0.20	0.44	**0.57**	0.67	0.75
Tox. rate	0.05	**0.10**	0.11	0.30	0.34	0.05	0.07	**0.22**	0.34	0.45
Utility	0.10	**0.37**	0.48	0.16	0.12	0.10	0.30	**0.13**	−0.01	−0.15
	Scenario 3					Scenario 4				
Mean eff.	0.20	0.21	**0.58**	0.77	0.77	0.20	0.20	0.20	0.22	**0**.**60**
Tox. rate	0.05	0.10	**0.18**	0.20	0.45	0.05	0.06	0.08	0.10	**0**.**24**
Utility	0.10	0.01	**0.22**	0.37	−0.13	0.10	0.08	0.04	0.02	**0**.**12**
	Scenario 5					Scenario 6				
Mean eff.	0.20	0.34	**0.68**	0.76	0.78	0.20	0.21	**0.72**	0.75	0.80
Tox. rate	0.05	0.12	**0.14**	0.35	0.45	0.05	0.06	**0.15**	0.24	0.28
Utility	0.10	0.10	**0.40**	0.06	−0.12	0.10	0.09	**0.42**	0.27	0.24
	Scenario 7					Scenario 8				
Mean eff.	0.20	0.24	0.41	**0.68**	0.78	0.20	0.23	0.32	**0.65**	0.79
Tox. rate	0.05	0.06	0.10	**0.12**	0.32	0.05	0.08	0.10	**0.32**	0.45
Utility	0.10	0.12	0.21	**0.44**	0.14	0.10	0.07	0.12	**0.01**	−0.11
	Scenario 9					Scenario 10				
Mean eff.	0.20	0.23	0.25	**0.72**	0.80	0.20	0.20	0.22	**0.54**	0.80
Tox. rate	0.05	0.06	0.08	**0.15**	0.34	0.05	0.06	0.08	**0.18**	0.20
Utility	0.10	0.11	0.09	**0.42**	0.12	0.10	0.08	0.06	**0.18**	0.40

The minimum effective doses are in boldface. The maximum utility doses are underlined.

To facilitate the comparison of the three methods, we calibrated $C_{PoC}$ to control the familywise type I error rate at 0.05 for each method under a null scenario with a flat efficacy curve of $\mu_{1}=\cdots =\mu_{5}=0.2$ and flat toxicity curve of $\pi_{1}=\cdots =\pi_{5}=0.05$ . For BQD, we set τ=0.5 for adaptive randomization, $C_{E}=0.7$ and $C_{T}=0.9$ in the admissible rule, and w=2 in the utility function (2.4). We use a slightly lower probability cutoff $C_{E}=0.7$ to ensure a higher probability of dropping futile doses.

For the ordinal efficacy endpoint, we assumed that $y_{i}$ takes an ordinal value of {0,1,2,3,4} . We generated $\left(x_{i},y_{i}\right)$ using a latent-variable approach. Specifically, we first generated a latent continuous efficacy variable, ${\tilde{y}}_{i}$ , and jointly generated a latent continuous toxicity variable, ${\tilde{x}}_{i}$ , which was based on a bivariate normal distribution (3.1) generated by replacing $y_{i}$ with ${\tilde{y}}_{i}$ in the equation. We then converted ${\tilde{y}}_{i}$ to an ordinal value of $y_{i}$ as follows: $y_{i}=k$ if $\gamma_{k}<{\tilde{y}}_{i}\leq \gamma_{k+1}$ , k=0,…,4 , where $\gamma_{k},\ldots ,\gamma_{k+1}$ were cutoffs used to bin ${\tilde{y}}_{i}$ to categories with $\gamma_{0}\equiv -\infty$ and $\gamma_{5}\equiv \infty$ . We converted ${\tilde{x}}_{i}$ to binary $x_{i}$ as described above. We considered six scenarios (Table 2), which were constructed by generating $\left({\tilde{x}}_{i},{\tilde{y}}_{i}\right)$ based on scenarios 1,3,4,5,7, and 8 in Table 1 for the continuous efficacy endpoint. We chose $\gamma_{0},\ldots ,\gamma_{5}$ to vary $p_{k}=\mathrm{Pr}(y_{i}=k),k=0,\ldots ,4$ . Table S2 in the Supplemental Material provides the values of $\left(p_{1},\ldots ,p_{5}\right)$ for the six scenarios. The maximum sample size was N=240 , enrolled in S=5 stages with cohort size $c_{1}=120$ , $c_{2}=\cdots =c_{5}=30$ . We used slightly different sample sizes and cohort sizes from these for the continuous endpoint to ensure reasonable power ( 80%−90% ) to establish the PoC in most scenarios.

**Table 2.** Simulation scenarios for the ordinal efficacy endpoint.

	Dose levels					Dose levels				
	0	1	2	3	4	0	1	2	3	4
	Scenario 1					Scenario 2				
Mean eff.	0.80	**1.55**	1.80	1.92	2.00	0.80	0.83	**1.56**	1.93	1.94
Tox. rate	0.05	**0.10**	0.11	0.30	0.34	0.05	0.10	**0.18**	0.20	0.45
Utility	0.60	**1.15**	1.36	0.72	0.64	0.60	0.43	**0.84**	1.13	0.14
	Scenario 3					Scenario 4				
Mean eff.	0.80	0.80	0.80	0.83	**1.60**	0.80	1.08	**1.76**	1.92	1.96
Tox. rate	0.05	0.06	0.08	0.10	**0.24**	0.05	0.12	**0.14**	0.35	0.45
Utility	0.60	0.56	0.48	0.43	**0.64**	0.60	0.60	**1.20**	0.52	0.16
	Scenario 5					Scenario 6				
Mean eff.	0.80	0.87	1.22	**1.75**	1.95	0.80	0.86	1.04	**1.70**	1.98
Tox. rate	0.05	0.06	0.10	**0.12**	0.32	0.05	0.08	0.10	**0.32**	0.45
Utility	0.60	0.63	0.82	**1.27**	0.67	0.60	0.54	0.64	**0.42**	0.18

The minimum effective doses are in boldface. The maximum utility doses are underlined.

For the binary efficacy endpoint, we generated $y_{i}$ using a latent-variable approach similar to that used for the ordinary efficacy endpoint . We first generated latent continuous variables, $\left({\tilde{x}}_{i},{\tilde{y}}_{i}\right)$ , based on a bivariate normal distribution, (3.1), and then converted ${\tilde{y}}_{i}$ to a binary value, $y_{i}$ , as follows: $y_{i}=1$ if $\Phi^{-1}({\tilde{y}}_{i})>1-\pi_{E,j}$ , otherwise $y_{i}=0$ , where $\pi_{E,j}$ is the efficacy rate for $d_{j}$ . Again, we considered six scenarios (Table 3), which were constructed by generating $\left({\tilde{x}}_{j},{\tilde{y}}_{i}\right)$ based on scenarios 1,3,4,5,7, and 8 of the continuous efficacy endpoint (Table 1). The maximum sample size was N=160 , enrolled in S=5 stages with cohort size $c_{1}=80$ and $c_{2}=\cdots =c_{5}=20$ to ensure reasonable power ( 80%−90% ) to detect the PoC in most scenarios. Because MCP-Mod and ANOVA cannot handle binary endpoints, we compared BQD with an MCP-Mod extension^24^ (denoted as MCP-Mod-B) that was proposed to handle a binary efficacy endpoint. MCP-Mod-B targets the MED and thus was used as a comparator to evaluate MED selection.

**Table 3.** Simulation scenarios for the binary efficacy endpoint.

	Dose levels					Dose levels				
	0	1	2	3	4	0	1	2	3	4
	Scenario 1					Scenario 2				
Eff. rate	0.20	**0.39**	0.45	0.48	0.50	0.20	0.21	**0.39**	0.48	0.48
Tox. rate	0.05	**0.10**	0.11	0.30	0.34	0.05	0.10	**0.18**	0.20	0.45
Utility	0.15	**0.29**	0.34	0.18	0.16	0.15	0.11	**0.21**	0.28	0.03
	Scenario 3					Scenario 4				
Eff. rate	0.20	0.20	0.20	0.21	**0.40**	0.20	0.27	**0.44**	0.48	0.49
Tox. rate	0.05	0.06	0.08	0.10	**0.24**	0.05	0.12	**0.14**	0.35	0.45
Utility	0.15	0.14	0.12	0.11	**0.16**	0.15	0.15	**0.30**	0.13	0.04
	Scenario 5					Scenario 6				
Eff. rate	0.20	0.22	0.30	**0.44**	0.49	0.20	0.22	0.26	**0.43**	0.50
Tox. rate	0.05	0.06	0.10	**0.12**	0.32	0.05	0.08	0.10	**0.32**	0.45
Utility	0.15	0.16	0.20	**0.32**	0.17	0.15	0.14	0.16	**0.11**	0.05

The minimum effective doses are in boldface. The maximum utility doses are underlined.

Notice that, both MCP-Mod and ANOVA consider only efficacy and the target MED and were used as comparators to evaluate the performance of BQD in MED selection for continuous, ordinal and binary efficacy endpoint. There is no existing dose-ranging method targeting MUD. We will thus only present the simulation results in MUD selection for our proposed method to demonstrate the advantage of the BQD design to select multiple target doses. We believe that this MUD selection step can have great potential to be further developed in dose-ranging designs.

We performed 10,000 simulations for each scenario. The operating characteristics of the designs were evaluated in three aspects: (1) the power to detect the dose-response (i.e. the power for the PoC); (2) the correct selection of the MED and MUD; and (3) patient allocation to treatment arms. Specifically, for the second aspect, we will evaluate the percentage of correct selection of the MED ( ${\mathrm{PCS}}_{\mathrm{MED}}$ ) and the MUD ( ${\mathrm{PCS}}_{\mathrm{MUD}}$ ), defined as the percentage of simulated trials that correctly selected the true MED and the true MUD, respectively. To facilitate the application of the proposed method, we have created a Github repository for the R codes to reproduce the results in the article, which can be found in https://github.com/FTBIOS666/Bayesian-quasi-likelihood-dose-ranging-design.

### 3.2. Results

#### 3.2.1. Results for continuous efficacy endpoint

**Figure 2.**
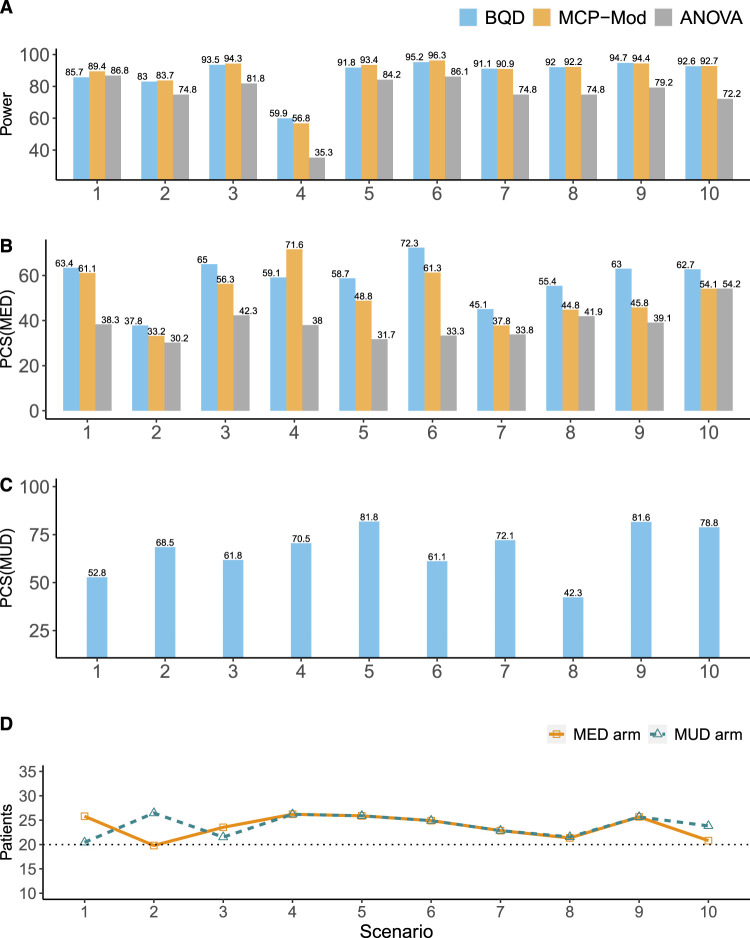
Simulation results for the continuous efficacy endpoint. (A) The power for establishing the PoC. (B) The percentages of the correct selection of the MED ( % ). (C) The percentages of the correct selection of the MUD ( % ). (D) The percentages of patients allocated to the MED or MUD; the horizontal dotted black line denotes the percentage of patients in each arm in fixed one-stage designs (i.e. 20% ), such as MCP-Mod. BQD: Bayesian quasi-likelihood dose-ranging design; ANOVA: analysis of variance; PoC: proof-of-concept; MED: minimum effective dose; MUD: maximum utility dose; MCP-Mod: multiple comparison test and modeling process.

Figure 2 shows the simulation results for the continuous efficacy endpoint. Figure 2(A) displays the power for establishing the PoC. The power BQD had for establishing the PoC was similar to that of MCP-Mod and up to 24.6% (mean, 12.95% ) greater than that of ANOVA. As described above, the posterior probability cutoff for establishing the PoC (i.e. $C_{PoC}$ ) was calibrated such that each method controlled the familywise type I error rate at 0.05 under the null scenario of a flat efficacy curve.

In terms of the percentage of correct selection of the MED ( ${\mathrm{PCS}}_{\mathrm{MED}}$ ), ANOVA generally had the lowest ${\mathrm{PCS}}_{\mathrm{MED}}$ values (see Figure 2(B)). This is consistent with previous simulation studies.^2,9^ BQD had similar performance as MCP-Mod when the dose–efficacy model was correctly specified for the latter. If the dose–efficacy model was not correctly specified, BQD outperformed MCP-Mod because BQD did not impose any parametric assumptions on the dose-efficacy curve. Specifically, in scenarios 1–4, in which the dose–efficacy model was correctly specified for MCP-Mod, MCP-Mod and BQD performed similarly: the two designs had similar ${\mathrm{PCS}}_{\mathrm{MED}}$ values in scenarios 1 and 2, and whereas BQD had a higher ${\mathrm{PCS}}_{\mathrm{MED}}$ in scenario 3, MCP-Mod had a higher ${\mathrm{PCS}}_{\mathrm{MED}}$ in scenario 4. In scenarios 5–10, in which the dose–efficacy model was arbitrarily generated and not necessarily correctly specified, BQD had higher ${\mathrm{PCS}}_{\mathrm{MED}}$ values than MCP-Mod did. For example, in scenarios 5 and 6, the ${\mathrm{PCS}}_{\mathrm{MED}}$ values of BQD were 9.9% and 11% higher, respectively, than those of MCP-Mod. Figure 2(C) displays the percentage of correct selection of the MUD ( ${\mathrm{PCS}}_{\mathrm{MUD}}$ ). Overall, BQD showed robust performance with eight out of 10 scenarios having ${\mathrm{PCS}}_{\mathrm{MUD}}$ values around or above 60% .

In terms of patient allocation, BQD generally outperformed MCP-Mod and ANOVA, allocating higher percentages of patients to the MED and MUD arms (Figure 2(D)). Because ANOVA and MCP-Mod utilize equal randomization to five arms, the percentages of patients they allocate to the MED and MUD arms are both fixed at 20% . In contrast, BQD employs adaptive randomization and assigns higher percentages of patients to the MED and MUD arms in most scenarios.

#### 3.2.2. Results for ordinal efficacy endpoint

**Figure 3.**
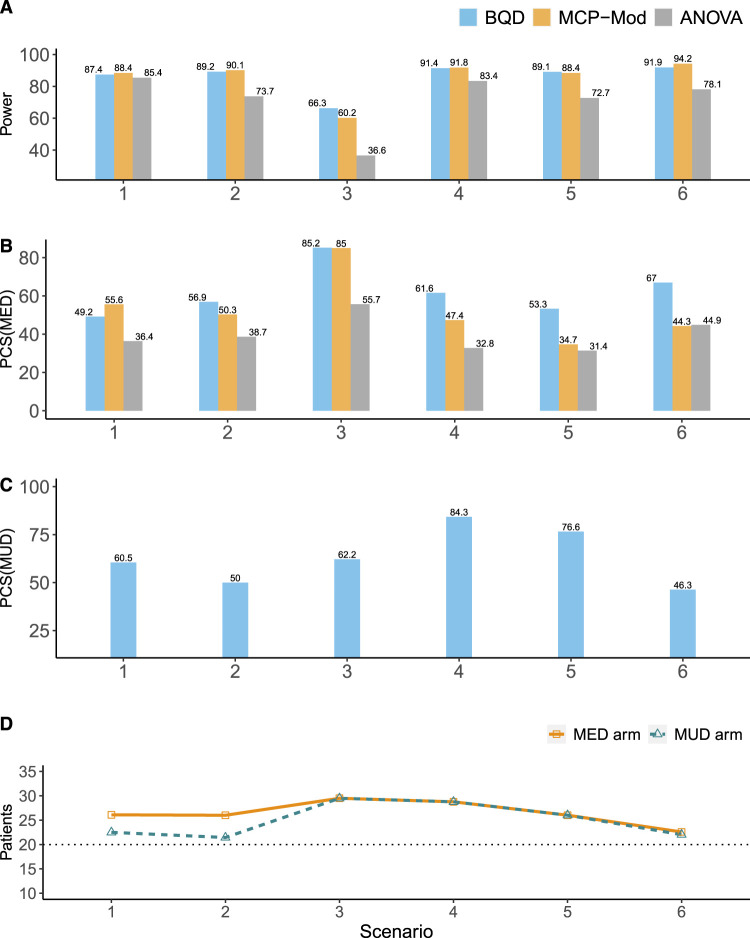
Simulation results for the ordinal efficacy endpoint. (A) The power for establishing the PoC. (B) The percentages of the correct selection of the MED ( % ). (C) The percentages of the correct selection of the MUD ( % ). (D) The percentages of patients allocated to the MED or MUD; the horizontal dotted black line denotes the percentage of patients in each arm in fixed one-stage designs (i.e. 20% ), such as MCP-Mod. BQD: Bayesian quasi-likelihood dose-ranging design; ANOVA: analysis of variance; PoC: proof-of-concept; MED: minimum effective dose; MUD: maximum utility dose; MCP-Mod: multiple comparison test and modeling process.

Figure 3 shows the simulation results for the ordinal efficacy endpoint. Similar to the results for the continuous endpoint, the power BQD had for establishing the PoC was similar to that of MCP-Mod, and both BQD and MCP-Mod outperformed ANOVA (Figure 3(A)). In terms of selecting the MED (see Figure 3(B)), ANOVA had the worst performance with the lowest ${\mathrm{PCS}}_{\mathrm{MED}}$ . BQD performed similarly to MCP-Mod when the dose–efficacy model was correctly specified for the latter (e.g. scenarios 1–3) and outperformed MCP-Mod when the dose–efficacy model was not correctly specified for the latter (e.g. scenarios 4–6). For example, in scenarios 4–6, the ${\mathrm{PCS}}_{\mathrm{MED}}$ values of BQD were 14.4% , 18.8% , and 22.7% higher, respectively, than those of MCP-Mod. In addition, BQD again showed robust performance in selecting the MUD (Figure 3(C)). In four of six scenarios (i.e. scenarios 1 and 3– 6 ), BQD resulted in ${\mathrm{PCS}}_{\mathrm{MUD}}$ values around or above 60% . In terms of patient allocation, BQD generally outperformed MCP-Mod and ANOVA, allocating higher percentages of patients to the MED and MUD arms (Figure 3(D)).

#### 3.2.3. Results for binary efficacy endpoint

**Figure 4.**
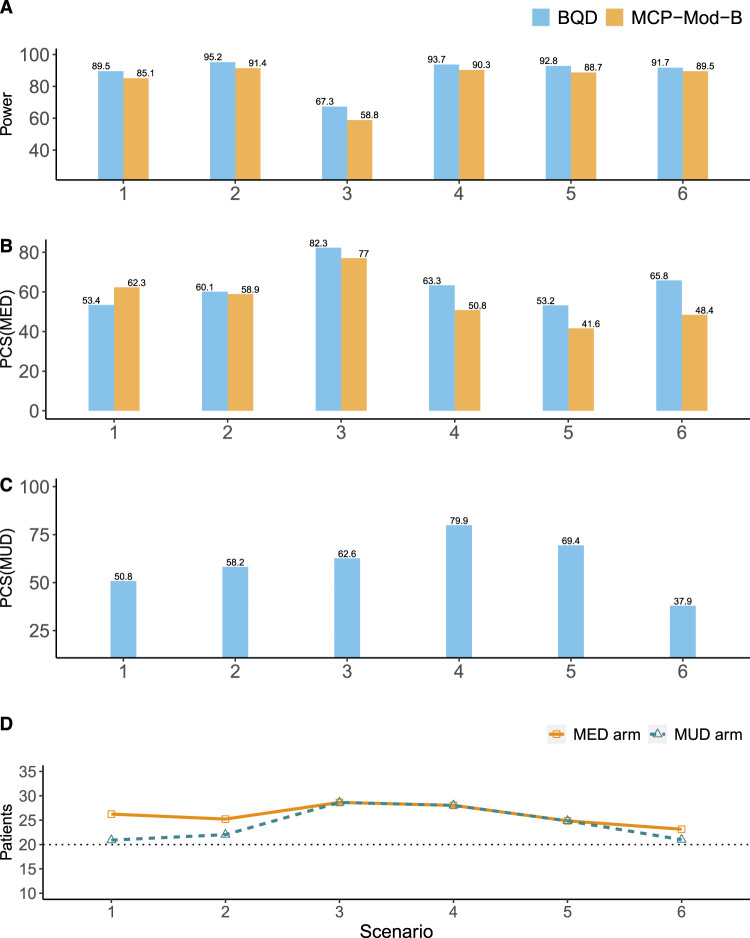
Simulation results for the binary efficacy endpoint. (A) The power for establishing the PoC. (B) The percentages of the correct selection of the MED ( % ). (C) The percentages of the correct selection of the MUD ( % ). (D) The percentages of patients allocated to the MED or MUD; the horizontal dotted black line denotes the percentage of patients in each arm in fixed one-stage designs (i.e. 20% ), such as MCP-Mod. BQD: Bayesian quasi-likelihood dose-ranging design; ANOVA: analysis of variance; PoC: proof-of-concept; MED: minimum effective dose; MUD: maximum utility dose; MCP-Mod: multiple comparison test and modeling process.

The results for the binary efficacy endpoint are generally similar to those for the continuous and ordinal endpoints, but the advantage of BQD is more pronounced. As shown in Figure 4(A) and (D), compared with MCP-Mod-B, BQD had higher power for establishing the PoC in all six scenarios. In addition, in most scenarios, BQD yielded higher selection percentages of ${\mathrm{PCS}}_{\mathrm{MED}}$ values than MCP-Mod-B did and resulted in ${\mathrm{PCS}}_{\mathrm{MED}}$ values around or above 60% . Also, BQD generally allocated higher percentages of patients to the MED and MUD arms than MCP-Mod-B.

### 3.3. Sensitivity analysis

We conducted sensitivity analyses for different number of dose levels (i.e. J=4 and J=7 ) as shown in Supplemental Tables S4 and S5 and Figures S1 and S2; different degrees of correlation between efficacy and toxicity endpoints (i.e. ρ=0.1,0.5 , and 0.7 ) as displayed in Supplemental Figure S3; and different adaptive allocation rules (i.e. τ=1 and τ=0 ) as shown in Supplemental Figure S4. The results (see Supplemental Material) are similar to those described above, showing that the BQD design is generally robust to these design parameters.

The performance of BQD is similar to or better than that of MCP-Mod, suggesting that the quasi-Bernoulli likelihood approach is highly efficient. To further investigate its efficiency, we compared BQD (based on the quasi-Bernoulli likelihood) with a version of BQD based on the true likelihood (denoted by BQD-L). The only difference between BQD and BQD-L is that the latter uses the true likelihood; all other decision rules are the same for the two designs. Thus, BQD-L provides the upper bound of the performance. Figure S5 in the Supplemental Material shows that BQD and BQD-L have similar performance, with similar power for establishing the PoC and similar accuracy in identifying the MED and MUD, further confirming the high efficiency of the quasi-Bernoulli likelihood approach.

## 4. Discussion

We propose a Bayesian quasi-likelihood dose-ranging design that handles various types of efficacy endpoints (e.g. binary, ordinal, and semi-continuous endpoints) and explicitly incorporates benefit–risk assessment into the decision of dose assignment and selection. Our design utilizes a utility function as a measure of benefit–risk tradeoff and adaptively assigns patients to doses based on the doses’ likelihood of being the MED and MUD. The design takes a group-sequential approach. At each interim, the doses that are deemed overly toxic or futile are dropped. The simulation study shows that the BQD design is robust and has competitive performance in selecting the MED. Additionally, it includes an extra feature for further MUD selection.

The BQD design models the toxicity and efficacy at each dose locally without making any parametric assumption on the shape of dose–efficacy or dose–toxicity curves. This makes the design simple (with closed-form posteriors) and more robust than MCP-Mod. Simulation shows that this nonparametric approach does not compromise the efficiency of the BQD design. This is because the isotonic regression utilizes efficacy and toxicity data across dose levels, which improves the efficiency of estimation and decision making. In addition, the design’s adaptive randomization and Bayesian decision rules enable more efficient patient allocation and provide extra flexibility to calibrate the design to improve the operating characteristics.

To accommodate various types of efficacy endpoints and streamline posterior calculations, we adopt a hybrid approach, where we model toxicity using its true likelihood while modeling efficacy using quasi-Bernoulli likelihood. It’s crucial to emphasize that our design does not mandate the use of the hybrid approach; the true likelihood of the efficacy endpoint can be employed. However, this alternative necessitates distinct models for different types of endpoints (e.g. the Bernoulli model for binary endpoints, the normal model for continuous endpoints, and the multinomial model for categorical endpoints) and involves more complex computations. Both our simulation and previous research^28,27,29^ demonstrate that the quasi-likelihood is highly efficient and yields desirable operating characteristics.

The BQD design can be extended in various ways. In some trials, the efficacy endpoint is the time to the event. In this case, a quasi-Bernoulli or quasi-normal likelihood approach cannot be used. We may use a piecewise exponential model and modify the utility along the lines of Murray et al.^34^ In addition, the BQD design assumes that the efficacy endpoint is quickly ascertainable for adaptive randomization and group sequential decisions. If this assumption does not hold, we may use imputation methods^32,35^ or likelihood approximation methods^36^ to facilitate real-time decision making.

## Supplemental Material

sj-pdf-1-smm-10.1177_09622802241239268 - Supplemental material for A Bayesian quasi-likelihood design for identifying the minimum effective dose and maximum utility dose in dose-ranging studies Supplemental material, sj-pdf-1-smm-10.1177_09622802241239268 for A Bayesian quasi-likelihood design for identifying the minimum effective dose and maximum utility dose in dose-ranging studies by Feng Tian, Ruitao Lin, Li Wang and Ying Yuan in Statistical Methods in Medical Research
