# Supplementary material for: A Bayesian quasi-likelihood design for identifying the minimum effective dose and maximum utility dose in dose-ranging studies
Source: Stat Methods Med Res. 2024 Apr 4;33(6):931–44. doi: 10.1177/09622802241239268 (PMC11162096; doi:10.1177/09622802241239268)
Supplement: sj-pdf-1-smm-10.1177_09622802241239268 - Supplemental material for A Bayesian quasi-likelihood design for identifying the minimum effective dose and maximum utility dose in dose-ranging studies [file sj-pdf-1-smm-10.1177_09622802241239268.pdf]

Supplementary Materials for "A Bayesian quasi-likelihood design for identifying the minimum effective dose and maximum utility dose in dose-ranging studies"

Feng Tian[1], Ying Yuan[1*], Ruitao Lin[1], and Li Wang[2]

[1] Department of Biostatistics, The University of Texas MD Anderson Cancer Center

Houston, Texas 77030, U.S.A.

[2] Department of Statistics, AbbVie Inc.

North Chicago, Illinois, U.S.A. *Corresponding author: yyuan@mdanderson.org

The following supporting information is contained in this supplementary materials:

Table S1. Models used to generate scenarios 1-4 for the continuous efficacy endpoint.

Table S2. Probabilities of ordinal scores used to generate the mean efficacy values for the ordinal efficacy endpoint.

Table S3. Average percentages of patients allocated to each dose level.

Table S4. Simulation scenarios with 4 dose levels.

Table S5. Simulation scenarios with 7 dose levels.

Table S6. Algorithm to obtain posterior probabilities of each dose being MED or MUD.

Figure S1. Results of the simulation scenarios with 4 dose levels.

Figure S2. Results of the simulation scenarios with 7 dose levels.

Figure S3. Sensitivity analysis for the correlation between toxicity and efficacy endpoints.

Figure S4. Sensitivity analysis for different adaptive allocation rules.

Figure S5. Evaluation of the efficiency of the quasi-Bernoulli likelihood approach used by the Bayesian quasi-likelihood dose-ranging (BQD) design.

1 Simulation setting for the continuous efficacy endpoint

Table S1: Models used to generate scenarios 1-4 for the continuous efficacy endpoint.

Model	Specification of mean response
$E_{\max}$	$0.2 + 0.75d/(1 + d)$
Linear in log-dose	$0.2 + 0.34\log(d + 1)$
Logistic	$0.2 + 0.57/\{1 + \exp[(1.84 - d)/0.23]\}$
Exponential	$0.2 + 0.1\exp[(d/0.31) - 12.51]$

Note: $d = \{0, 1, 2, 3, 4\}$

2 Simulation setting for the ordinal efficacy endpoint

Table S2: Probabilities of ordinal scores used to generate the mean efficacy values for the ordinal efficacy endpoint.

	Ordinal score				
	0	1	2	3	4
Mean eff.	p_1	p_2	p_3	p_4	p_5
0.80	0.6200	0.2000	0.0400	0.0400	0.1000
0.83	0.6200	0.1900	0.0400	0.0400	0.1100
0.86	0.6050	0.2000	0.0400	0.0400	0.1150
0.87	0.6025	0.2000	0.0400	0.0400	0.1175
1.04	0.5575	0.2000	0.0400	0.0500	0.1525
1.08	0.5475	0.2000	0.0400	0.0500	0.1625
1.22	0.5125	0.2000	0.0400	0.0500	0.1975
1.55	0.4500	0.1500	0.0500	0.1000	0.2500
1.56	0.4475	0.1500	0.0500	0.1000	0.2525
1.60	0.4375	0.1500	0.0500	0.1000	0.2625
1.70	0.4125	0.1500	0.0500	0.1000	0.2875
1.75	0.3875	0.1500	0.0500	0.1500	0.2625
1.76	0.3925	0.1500	0.0500	0.1200	0.2875
1.80	0.3825	0.1500	0.0500	0.1200	0.2975
1.92	0.3525	0.1500	0.0500	0.1200	0.3275
1.93	0.3925	0.1000	0.0500	0.1000	0.3575
1.94	0.3900	0.1000	0.0500	0.1000	0.3600
1.95	0.3875	0.1000	0.0500	0.1000	0.3625
1.96	0.3850	0.1000	0.0500	0.1000	0.3650
1.98	0.3425	0.1500	0.0500	0.1000	0.3575
2.00	0.3750	0.1000	0.0500	0.1000	0.3750

3 Simulation results for patient allocation

Table S3: Average percentages of patients allocated to each dose level. The minimum effective doses are in boldface. The maximum utility doses are underlined.

	Dose levels					Dose levels				
	0	1	2	3	4	0	1	2	3	4
	Scenario 1					Scenario 2				
Mean eff.	0.20	**0.57**	0.70	0.76	0.80	0.20	0.44	**0.57**	0.67	0.75
Tox. rate	0.05	**0.10**	0.11	0.30	0.34	0.05	0.07	**0.22**	0.34	0.45
Utility	0.10	**0.37**	0.48	0.16	0.12	0.10	0.30	**0.13**	-0.01	-0.15
Patients (%)	23.50	**25.94**	20.18	14.99	15.38	23.89	26.39	**19.88**	16.36	13.48
	Scenario 3					Scenario 4				
Mean eff.	0.20	0.21	**0.58**	0.77	0.77	0.20	0.20	0.20	0.22	**0.60**
Tox. rate	0.05	0.10	**0.18**	0.20	0.45	0.05	0.06	0.08	0.10	**0.24**
Utility	0.10	0.01	**0.22**	0.37	-0.13	0.10	0.08	0.04	0.02	**0.12**
Patients (%)	23.42	18.53	**23.62**	21.36	13.06	23.62	16.07	15.96	18.05	**26.30**
	Scenario 5					Scenario 6				
Mean eff.	0.20	0.34	**0.68**	0.76	0.78	0.20	0.21	**0.72**	0.75	0.80
Tox. rate	0.05	0.12	**0.14**	0.35	0.45	0.05	0.06	**0.15**	0.24	0.28
Utility	0.10	0.10	**0.40**	0.06	-0.12	0.10	0.09	**0.42**	0.27	0.24
Patients (%)	23.69	21.99	**25.85**	15.58	12.89	23.35	18.59	**24.77**	16.09	17.20
	Scenario 7					Scenario 8				
Mean eff.	0.20	0.24	0.41	**0.68**	0.78	0.20	0.23	0.32	**0.65**	0.79
Tox. rate	0.05	0.06	0.10	**0.12**	0.32	0.05	0.08	0.10	**0.32**	0.45
Utility	0.10	0.12	0.21	**0.44**	0.14	0.10	0.07	0.12	**0.01**	-0.11
Patients (%)	23.25	17.70	19.41	**22.75**	16.89	23.58	18.97	21.38	**21.50**	14.56
	Scenario 9					Scenario 10				
Mean eff.	0.20	0.23	0.25	**0.72**	0.80	0.20	0.20	0.22	**0.54**	0.80
Tox. rate	0.05	0.06	0.08	**0.15**	0.34	0.05	0.06	0.08	**0.18**	0.20
Utility	0.10	0.11	0.09	**0.42**	0.12	0.10	0.08	0.06	**0.18**	0.40
Patients (%)	23.45	17.12	17.19	**25.62**	16.63	23.26	15.89	16.19	**20.76**	23.90

4 Sensitivity analysis for the number of dose levels

4.1 4 doses

Table S4: Simulation scenarios with 4 dose levels. The minimum effective doses are in boldface. The maximum utility doses are underlined.

	Dose levels				Dose levels			
	0	1	2	3	0	1	2	3
	Scenario 1				Scenario 2			
Mean eff.	0.20	0.34	**0.68**	0.77	0.20	0.25	**0.59**	0.76
Tox. rate	0.05	0.15	**0.20**	0.32	0.05	0.20	**0.28**	0.45
Utility	0.10	0.04	**0.28**	0.13	0.10	-0.15	**0.03**	-0.14
	Scenario 3				Scenario 4			
Mean eff.	0.20	**0.59**	0.72	0.78	0.20	**0.66**	0.72	0.78
Tox. rate	0.05	**0.15**	0.20	0.32	0.05	**0.10**	0.14	0.18
Utility	0.10	**0.29**	0.32	0.14	0.10	**0.46**	0.44	42

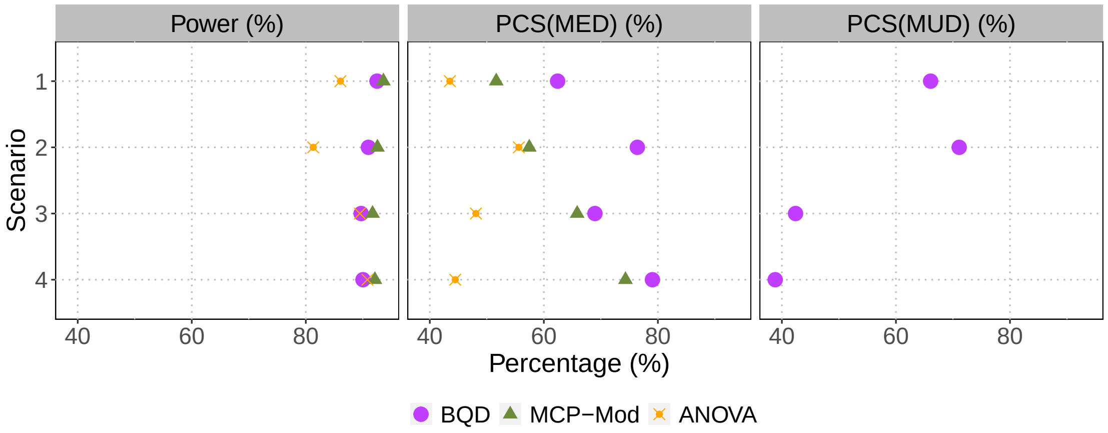

Figure S1: Results of the simulation scenarios with 4 dose levels. Left: The power for establishing the proof-of-concept (PoC). Middle: The percentages of the correct selection of the minimum effective dose (MED). Right: The percentages of the correct selection of the maximum utility dose (MUD).

4.2 7 doses

Table S5: Simulation scenarios with 7 dose levels. The minimum effective doses are in boldface. The maximum utility doses are underlined.

	Dose levels							Dose levels						
	0	1	2	3	4	5	6	0	1	2	3	4	5	6
	Scenario 1							Scenario 2						
Mean eff.	0.20	**0.62**	0.66	0.70	0.74	0.78	0.80	0.20	0.32	**0.59**	0.66	0.70	0.76	0.80
Tox. rate	0.05	**0.08**	0.12	0.15	0.19	0.23	0.27	0.05	0.15	**0.19**	0.22	0.28	0.32	0.45
Utility	0.10	**0.46**	0.42	0.40	0.36	0.32	0.26	0.10	0.02	**0.21**	0.22	0.14	0.12	-0.10
	Scenario 3							Scenario 4						
Mean eff.	0.20	0.23	0.28	**0.65**	0.76	0.78	0.80	0.20	0.23	0.25	0.27	**0.59**	0.76	0.80
Tox. rate	0.05	0.12	0.15	**0.18**	0.23	0.28	0.34	0.05	0.12	0.15	0.18	**0.23**	0.28	0.34
Utility	0.10	-0.01	-0.02	**0.29**	0.30	0.22	0.12	0.10	-0.01	-0.05	-0.09	**0.13**	0.20	0.12

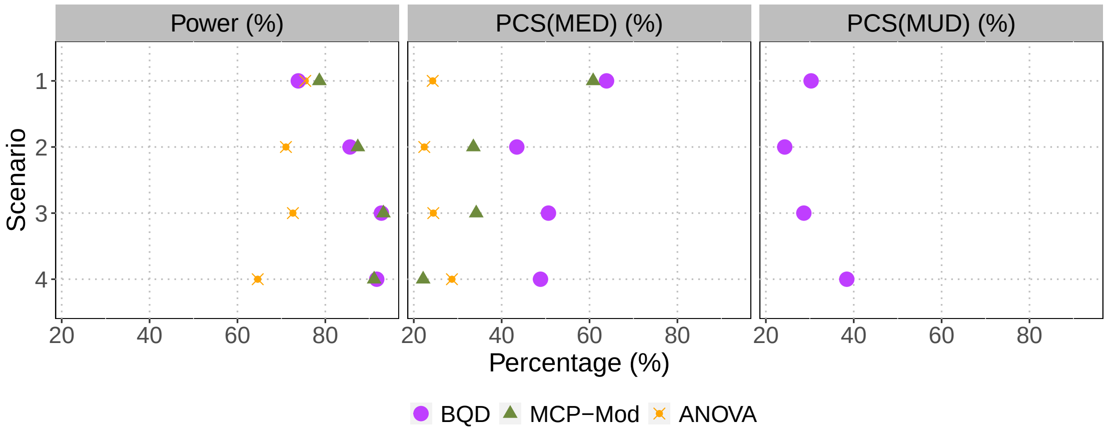

Figure S2: Results of the simulation scenarios with 7 dose levels. Left: The power for establishing the proof-of-concept (PoC). Middle: The percentages of the correct selection of the minimum effective dose (MED). Right: The percentages of the correct selection of the maximum utility dose (MUD).

5 Sensitivity analysis for the correlation between toxicity and efficacy endpoints

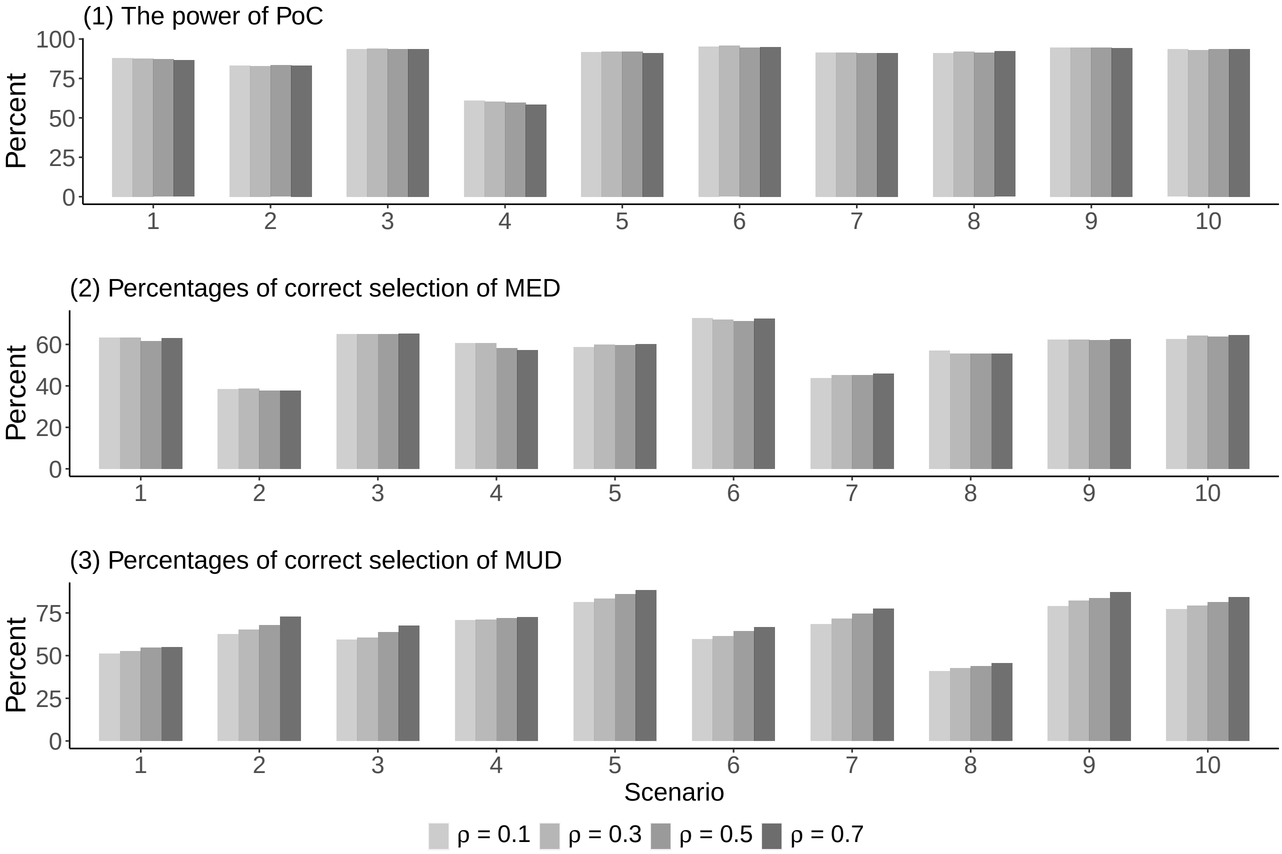

Figure S3: Proof-of-concept (PoC) power, minimum effective dose (MED) selection, and maximum utility dose (MUD) selection under different correlations between efficacy and safety endpoints for the proposed Bayesian quasi-likelihood dose-ranging (BQD) design.

6 Sensitivity analysis for different adaptive allocation rules

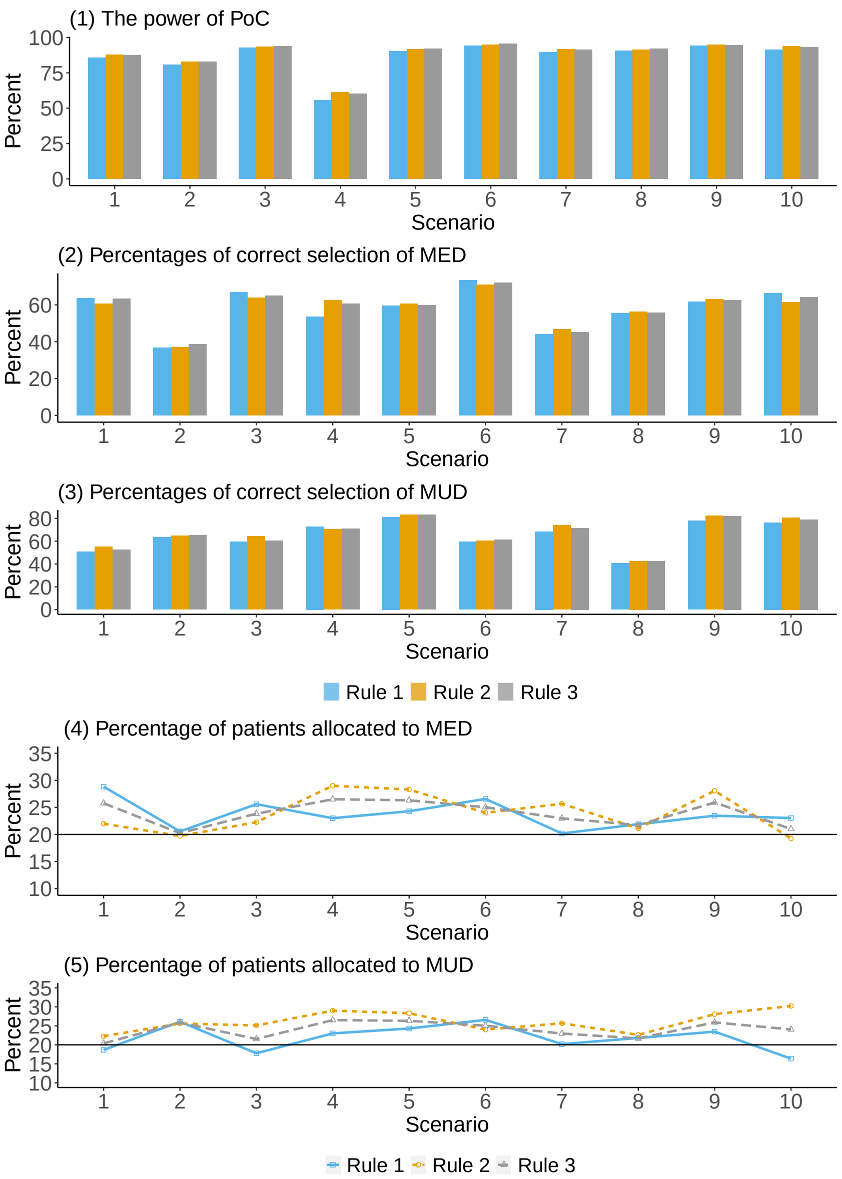

Figure S4: Proof-of-concept (PoC) power, minimum effective dose (MED) selection, and maximum utility dose (MUD) selection, and percentages of patients allocated to the MED or MUD arm under different adaptive allocation rules: rule 1 targets MED with $\tau = 1$, rule 2 targets MUD with $\tau = 0$, and rule 3 targets both MUD and MED with τ=0.5.

7 Evaluation of the efficiency of the quasi-Bernoulli likelihood approach used by the Bayesian quasi-likelihood dose-ranging (BQD) design

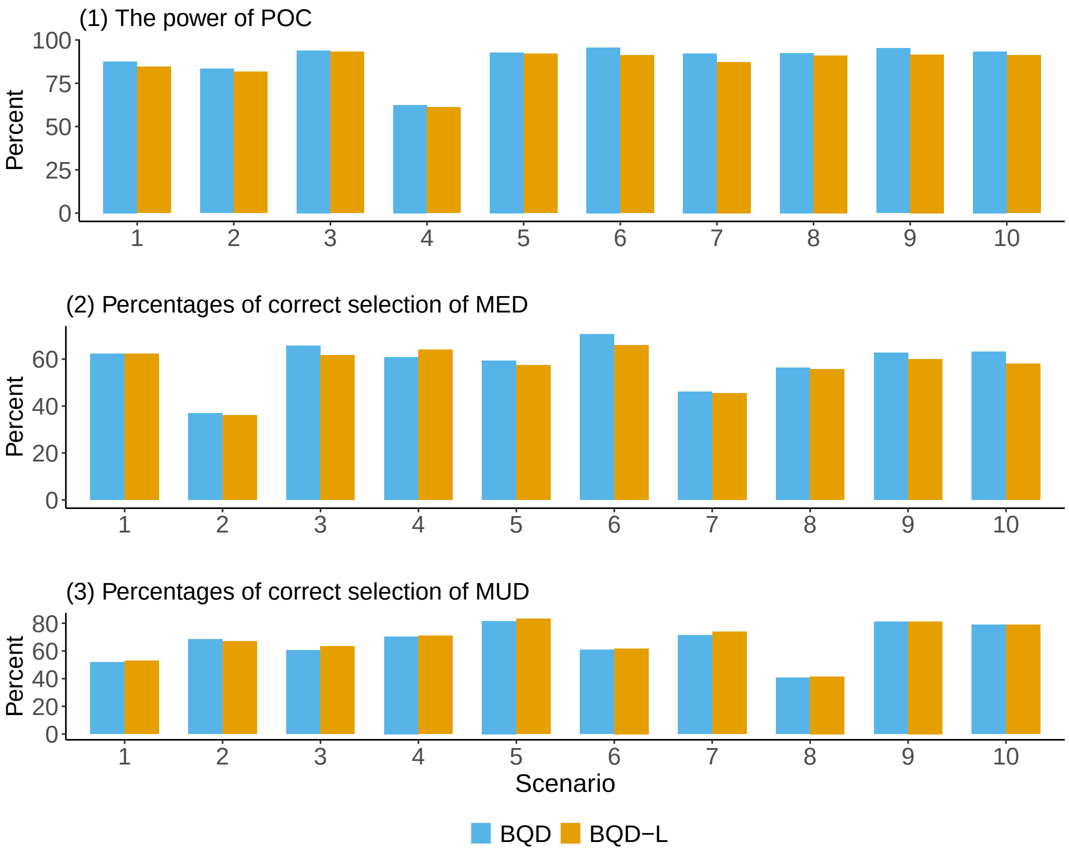

Figure S5: Comparison of proof-of-concept (PoC) power, minimum effective dose (MED) selection, and maximum utility dose (MUD) selection between BQD (based on the quasi-Bernoulli likelihood) and BQD-L (based on the true likelihood).

8 Algorithm to obtain posterior probabilities of each dose being MED or MUD

Table S6: Algorithm 1. Obtaining posterior probabilities that each dose being MED or MUD, i.e., $\{p_{1j}, j = 1, \ldots, J\}$ and $\{p_{2j}, j = 1, \ldots, J\}$

Require:

Observed toxicity and efficacy data D_n; sample size n; number of iterations T. In most cases, $T \geqslant 1000$ can guarantee a satisfactory performance.

1: Based on the observed data, update the isotonically transformed posterior distributions of toxicity rates $\pi_j|D_{(n)}, j = 1, ..., J$ and marginal efficacy $\mu_j|D_{(n)}, j = 1, ..., J$.

2: **for** $t = 1$ to T **do**

3: Draw a posterior sample of $\{\pi_j^{(t)}, j = 1, ..., J\}$ and $\{\mu_j^{(t)}, j = 1, ..., J\}$ via (1).

4: Obtain a posterior sample of the utility values $\{U_j^{(t)}, j = 1, ..., J\}$ by computing the corresponding utility values for each dose j, given $U_j^{(t)} = \mu_j^{(t)} - w\pi_j^{(t)}$.

5: Decide the location of MED $d_{MED}^{(t)}$ based on (2.8) and $\{\mu_j^{(t)}, j = 1, ..., J\}$, and decide the location of MUD $d_{MUD}^{(t)}$ based on (2.9) and $\{U_j^{(t)}, j = 1, \ldots, J\}$.

6: **end for**

7: **return** $\{d_{MED}^{(t)}, t = 1, \ldots, T\}$ and $\{d_{MUD}^{(t)}, t = 1, \ldots, T\}$.

8: After T iterations, calculate $p_{1j} = \Pr(d_{MED} = d_j|D_{(n)}) = \sum_{t=1}^{T} 1(d_{MED}^{(t)} = d_j)/T$ and $p_{2j} = \Pr(d_{MUD} = d_j|D_{(n)}) = \sum_{t=1}^{T} 1(d_{MUD}^{(t)} = d_j)/T$ for dose $j = 1, \ldots, J$.
